# Poking Around: A Comparative Study of Complications in Trans-rectal Versus Trans-perineal Prostate Biopsies

**DOI:** 10.7759/cureus.96991

**Published:** 2025-11-16

**Authors:** Jacob Chircop St John, Luke Borg, Alexander Carachi, Patrick Zammit

**Affiliations:** 1 Surgery, Mater Dei Hospital, Msida, MLT; 2 Urology, Mater Dei Hospital, Msida, MLT

**Keywords:** complications, prostate cancer, prostatitis, sepsis, transperineal biopsy, transrectal biopsy

## Abstract

Background

Trans-rectal prostate biopsy (TRPB) has historically been the commonest method for diagnosing prostate cancer but carries an increased risk of infection related to rectal flora. Trans-perineal prostate biopsy (TPPB), which avoids the rectal mucosa, is proposed to reduce infectious complications. This study compares post-biopsy infection rates and length of hospital stay (LOS) between TRPB and TPPB at Mater Dei Hospital, Malta.

Methods

A retrospective cohort study was conducted on all TRPBs performed in 2022 and all TPPBs performed in 2024 by urologists at Mater Dei Hospital. Outcomes analyzed were prostatitis, sepsis, and LOS. Sepsis was defined using Sepsis-3 criteria. Categorical values were analyzed using Chi-square and Fisher’s exact tests, and LOS was analyzed using Welch’s t-test and the Mann-Whitney U test. Statistical analysis was performed using Microsoft Excel (Redmond, WA, USA).

Results

Of 177 TRPB cases, 10 (5.6%) developed prostatitis compared with two of 231 TPPB cases (0.9%). This difference was statistically significant (χ²(1)=8.03, p=0.0046; Fisher’s p=0.0061). Median LOS for admitted patients was longer after TRPB (six days) than TPPB (five days), but this difference was not statistically significant (Welch t=0.898, p=0.507; Mann-Whitney U=15.5, p=0.415). No TPPB patient developed sepsis.

Conclusion

TPPB demonstrated significantly fewer infectious complications and eliminated sepsis compared with TRPB. These findings support the trans-perineal route as a safer standard of care.

## Introduction

Prostate cancer is a malignant tumour that originates in the epithelial cells of the prostate gland, most commonly arising from the peripheral zone. It represents the second most common solid tumour and the fifth leading cause of cancer-related death among men worldwide [1,2]. The mean age at diagnosis is approximately 66-67 years, and risk is influenced by age, family history, ethnicity, and genetic and metabolic factors [2-5]. The most frequently reported Gleason score at diagnosis is 3+4=7, reflecting an increasing trend toward detecting clinically significant but potentially curable disease [6,7].

Diagnosis continues to rely on histological sampling via prostate biopsy. Two primary approaches exist: the trans-rectal prostate biopsy (TRPB) and the trans-perineal prostate biopsy (TPPB). Both achieve comparable cancer detection rates [8]. However, TRPB introduces the biopsy needle through the rectal mucosa, where it may inoculate rectal flora into the prostate or bloodstream, resulting in prostatitis or sepsis [7]. In contrast, TPPB accesses the prostate through the perineal skin, which can be adequately sterilised, thereby substantially reducing infective complications [9-15].

In recent years, increasing antimicrobial resistance - particularly fluoroquinolone-resistant Escherichia coli - has heightened concern regarding post-biopsy sepsis following TRPB [9]. Consequently, many centres have transitioned toward TPPB as a safer alternative. This study compares post-biopsy infectious outcomes and length of hospital stay (LOS) between TRPB and TPPB performed at Mater Dei Hospital, Malta’s national tertiary referral centre for urological services.

Institutional experience

At Mater Dei Hospital, TRPB was historically the standard method for prostate biopsy, performed by urologists. A limited number of TPPB procedures were previously undertaken by a uro-radiologist specialising in urological interventions; however, these cases were excluded from this study to ensure cohort consistency. In mid-2023, following the return of a urologist trained in the trans-perineal approach, the department transitioned to routinely performing TPPB. The year 2024 represents the first full calendar year in which TPPB was adopted as the standard biopsy technique. This study therefore provides the first real-world institutional comparison of infectious outcomes before and after this change in practice.

## Materials and methods

This retrospective cohort study included 408 male patients who underwent prostate biopsy at Mater Dei Hospital between 1 January 2022 and 31 December 2024. Patients were divided into two groups based on biopsy approach: 177 underwent TRPB in 2022 and 231 underwent TPPB in 2024, reflecting the institutional technique transition described above. Biopsies performed by a uro-radiologist were excluded to ensure all procedures were performed by the same specialty team.

TRPB was performed under local anaesthesia (periprostatic block with 1% lignocaine). Patients received oral ciprofloxacin 500 mg one to two hours pre-procedure as antimicrobial prophylaxis.

TPPB was performed under local anaesthesia using a freehand trans-perineal technique. Patients received intravenous co-amoxiclav 1.2 g prior to biopsy as per the updated institutional protocol.

For each patient, clinical records, laboratory data, and discharge summaries were reviewed via the hospital’s electronic medical record system (ICM and Patient Dashboard). Data collected included biopsy type, occurrence of prostatitis or sepsis, length of stay, and microbiology results where available. Sepsis was defined according to Sepsis-3 criteria [8], assessed using SOFA score thresholds as described by Vincent et al. [9]. The components of the SOFA score used for this assessment are summarized in Table 1.

**Table 1:** Sequential (Sepsis-Related) Organ Function Assessment (SOFA) Score Adapted from Vincent et al. [9] Catecholamine dose in µg/kg/min, >1 hour. Glasgow Coma Scale ranges from 3–15 (3 = minimum, 15 = normal). Abbreviations: PaO₂/FiO₂ = partial pressure of oxygen/fraction of inspired oxygen, MAP = mean arterial pressure.

System / Score	0	1	2	3	4
Respiration: PaO₂/FiO₂ (mmHg) (kPa)	≥400 (53.3)	<400 (53.3)	<300 (40)	<200 (26.7) with respiratory support	<100 (13.3) with respiratory support
Coagulation: Platelets ×10³/µL	≥150	<150	<100	<50	<20
Liver: Bilirubin (mg/dL) (µmol/L)	<1.2 (20)	1.2–1.9 (20–32)	2.0–5.9 (33–101)	6.0–11.9 (102–204)	>12.0 (204)
Cardiovascular	MAP ≥70 mm Hg	MAP <70 mm Hg	Dopamine ≤5 or dobutamine (any dose)	Dopamine 5.1–15 or epinephrine ≤0.1, or norepinephrine ≤0.1¹	Dopamine >15 or epinephrine >0.1, or norepinephrine >0.1¹
Central Nervous System: Glasgow Coma Scale	15	13–14	10–12	6–9	<6
Renal: Creatinine (mg/dL) (µmol/L); Urine output (mL/day)	<1.2 (110)	1.2–1.9 (110–170)	2.0–3.4 (171–299)	3.5–4.9 (300–440); <500	>5.0 (440); <200

Statistical analyses were performed using Microsoft Excel (Redmond, WA, USA). Categorical variables were analysed using Chi-square or Fisher’s Exact Test, and length of stay using Welch’s t-test and Mann-Whitney U test. A p-value <0.05 was considered statistically significant.

As anonymised retrospective data were used, formal ethics approval was not required.

## Results

In the TRPB group (n = 177), 10 patients (5.55%) developed prostatitis requiring intravenous antibiotics. Two of these patients (20%) had positive blood cultures, yielding Escherichia coli and extended-spectrum β-lactamase (ESBL)-producing organisms. One patient met Sepsis-3 criteria for sepsis; however, no cases required transfer to the ICU or High Dependency Unit (HDU). The mean length of stay was 6.8 days (range, 5-12). Most patients received intravenous piperacillin/tazobactam, followed by discharge antibiotics such as co-amoxiclav or levofloxacin. In contrast, within the TPPB group (n = 231), two patients (0.87%) developed prostatitis. One patient cultured Pseudomonas aeruginosa and was treated with intravenous piperacillin/tazobactam followed by oral ciprofloxacin. No cases of sepsis or ICU/HDU transfer were observed in this cohort. The mean length of stay was five days (range, 3-7). A summary of these findings, including cohort sizes, rates of prostatitis and sepsis, and mean hospital stay, is presented in Table 2.

**Table 2:** Summary of results Table summarizing number of patients, percent of cohort developing prostatitis, percent of cohort developing sepsis and average length of stay for both trans-rectal prostate biopsy (TRPB) and trans-perineal prostate biopsy (TPPB) cohorts

Biopsy Method	Number of Patients	Prostatitis	Sepsis	Average Length of Admission (days)
TRPB	177	10 (5.55%)	1 (0.56%)	6.8
TPPB	231	2 (0.87%)	0 (0%)	5

The mean age of participants was comparable between groups, at 68.1 years (SD ± 7.9) for TRPB and 67.4 years (SD ± 8.2) for TPPB (Table 3). The incidence of prostatitis was significantly higher in the TRPB group than in the TPPB group (5.6% vs 0.9%), as shown in Table 4. Owing to the low number of events in the TPPB cohort, Fisher’s exact test was applied, confirming a statistically significant difference (Fisher’s p = 0.006; χ² = 8.03, p = 0.0046). The mean hospital stay was shorter in the TPPB group (five days) compared with the TRPB group (six days) (Table 5), although this difference did not reach statistical significance on either Welch’s t-test (t = 0.898, p = 0.507) or the Mann-Whitney U test (U = 15.5, p = 0.415).

**Table 3:** Baseline characteristics of study population Mean patient age was comparable between the trans-rectal prostate biopsy (TRPB) and trans-perineal prostate biopsy (TPPB) cohorts, with no statistically significant difference identified.

Variable	TRPB (n=177)	TPPB (n=231)
Age, mean ± SD (years)	68.1 ± 7.9	67.4 ± 8.2

**Table 4:** Comparison of infective complications between trans-rectal prostate biopsy (TRPB) and trans-perineal prostate biopsy (TPPB). The incidence of prostatitis was significantly higher following TRPB. Fisher’s Exact Test was used due to low expected counts in the TPPB group (Fisher p = 0.006; Chi-square χ² = 8.03, p = 0.0046).

Outcome	TRPB (n=177)	TPPB (n=231)	Statistical Result
Prostatitis, n (%)	10 (5.6%)	2 (0.9%)	χ²(1)=8.03, p=0.0046; Fisher p=0.0061

**Table 5:** Length of hospital stay (LOS) among admitted patients following trans-rectal prostate biopsy (TRPB) and trans-perineal prostate biopsy (TPPB). Although the median LOS was shorter for TPPB, the difference was not statistically significant on Welch’s t-test or Mann–Whitney U analysis.

Outcome	TRPB	TPPB	Statistical Result
LOS (days), median (range)	6 (5–12)	5 (3–7)	Welch t=0.898, p=0.507; Mann–Whitney U=15.5, p=0.415

## Discussion

Prostate biopsy remains the definitive diagnostic tool for confirming prostate cancer in men with elevated prostate-specific antigen (PSA) levels or suspicious imaging findings. The two most commonly employed approaches are the TRPB and the TPPB, both of which are performed under ultrasound guidance and provide comparable cancer detection rates [10]. Concerns surrounding post-procedural infectious complications have led to increasing international adoption of the trans-perineal approach in recent years.

In this study, we observed a significantly reduced incidence of prostatitis in patients undergoing TPPB compared with those undergoing TRPB (0.87% vs 5.55%), and no cases of sepsis occurred following TPPB, whereas one case was recorded following TRPB. Although the median length of hospital stay was shorter in the TPPB cohort, this difference did not reach statistical significance. These findings support the growing evidence that the trans-perineal route reduces infective complications when compared to the transrectal route.

Our observations are consistent with those of Huang et al. (2019), who reported lower rates of prostatitis and sepsis following TPPB compared with TRPB in a single-centre comparison [11]. Larger population-level studies have similarly demonstrated reduced infectious complications with TPPB, particularly in the context of rising fluoroquinolone resistance and increased prevalence of extended-spectrum β-lactamase (ESBL)-producing organisms [12,13]. The avoidance of rectal flora exposure is the most widely accepted explanation for this reduction in post-procedural infection risk. During TRPB, the biopsy needle traverses rectal mucosa, potentially inoculating enteric bacteria into the prostate or bloodstream [16,17]. In contrast, TPPB is performed through the perineal skin, which can be adequately sterilised, thereby reducing the likelihood of bacterial contamination [18,19].

This study has several limitations. As a retrospective single-centre analysis, it is subject to potential selection and information bias. The overall number of infection events was small, which limits the power to detect differences in rare complications. Additionally, the shift in biopsy practice coincided with updated antimicrobial prophylaxis protocols, which may have contributed to the observed reduction in infection rates. Nonetheless, the consistency of our findings with published literature supports their clinical relevance.

## Conclusions

This study provides real-world data from a national tertiary urological centre during the transition from transrectal to trans-perineal prostate biopsy. The adoption of TPPB was associated with a significantly lower rate of post-biopsy prostatitis and the elimination of sepsis in this cohort, without prolonging hospital stay. These findings support the trans-perineal approach as a safer biopsy method with respect to infectious complications. Further prospective, multi-centre evaluation is warranted to confirm these results and to assess long-term outcomes and implementation across broader clinical settings.
